# The efficacy and safety of irinotecan ± bevacizumab compared with oxaliplatin ± bevacizumab for metastatic colorectal cancer: A meta-analysis

**DOI:** 10.1097/MD.0000000000017384

**Published:** 2019-09-27

**Authors:** Jiali Dai, Yuetong Chen, Yang Gong, Jingsun Wei, Xiaowen Cui, Hualin Yu, Wenjing Zhao, Dongying Gu, Jinfei Chen

**Affiliations:** a Department of Oncology, Nanjing First Hospital, Nanjing Medical University; b Cancer Center, Taikang Xianlin Drum Tower Hospital, Nanjing University; c Collaborative Innovation Center for Cancer Personalized Medicine, Nanjing Medical University, Nanjing, China.

**Keywords:** bevacizumab, chemotherapy, colorectal cancer, irinotecan, meta-analysis, oxaliplatin

## Abstract

**Background:**

Irinotecan (IRI)-based and oxaliplatin (OXA)-based regimens are available for the treatment of metastatic colorectal cancer (mCRC). Several studies have published inconsistent results in their comparisons of the efficacy and toxicity of IRI ± bevacizumab and OXA ± bevacizumab. This meta-analysis was performed to evaluate the efficacy and safety of these 2 regimens in patients with mCRC.

**Methods:**

We searched several databases to identify relevant studies, including PubMed, EMBASE, and the Cochrane Controlled Trials Register. The primary endpoints were overall survival (OS) and time to progression (TTP). The secondary comparisons were overall response rate (ORR) and toxicity. In addition, the hazard ratio (HR) or risk ratio (RR) values with their corresponding 95% confidence intervals (CIs) were extracted from these studies.

**Results:**

Pooled data of 13 studies demonstrated no significant differences in OS (HR = 0.96, 95% CI: 0.86–1.08, *P* = .53) and TTP (HR = 0.88, 95% CI: 0.72–1.08, *P* = .24) between the 2 groups. However, the ORR (RR = 0.87, 95% CI: 0.78–0.97, *P* = .02) was clearly improved in the OXA ± bevacizumab arm. Higher incidences of grade 3/4 nausea (RR = 1.63, 95% CI: 1.28–2.07, *P* < .001), vomiting (RR = 1.40, 95% CI: 1.09–1.81, *P* = .01), diarrhea (RR = 1.44, 95% CI: 1.23–1.70, *P* < .001), and anemia (RR = 4.13, 95% CI: 2.75–6.22, *P* < .001) were observed in the IRI group. However, the incidences of grade 3/4 neutropenia (RR = 0.75, 95% CI: 0.68–0.83, *P* < .001), thrombocytopenia (RR = 0.43, 95% CI: 0.26–0.73, *P* = .002), and paresthesia/neurological disturbances (RR = 0.04, 95% CI: 0.02–0.07, *P* < .001) were higher in the OXA group.

**Conclusion:**

This meta-analysis confirmed that the OXA ± bevacizumab regimen as a maintenance therapy significantly improved the ORR in patients with mCRC. Exhibiting strong efficacy and safety, the OXA and OXA plus bevacizumab regimens are preferred as first-line treatments for mCRC.

## 1 Introduction

Colorectal cancer (CRC) is the second most common cancer in females and the third most common cancer in males and is also the fourth leading cause of cancer-related death worldwide.^[1]^ The incidence of CRC has an upward trend due to the dietary and lifestyle changes that have occurred over the last several decades.^[2]^ Most patients are already in an advanced stage at diagnosis; hence, they cannot receive curative (R0) surgical resection.^[2]^ Recently, chemotherapy drugs have deeply changed the prognosis of these patients. Although chemotherapy drugs are associated with some clinical benefits, the treatment efficacy is limited.

Irinotecan (IRI) and oxaliplatin (OXA) are the most common cytotoxic chemotherapy drugs used in the treatment of metastatic colorectal cancer (mCRC), as they have a relatively preferable antitumor activity.^[3]^ IRI is a semisynthetic derivative of camptothecin and is available for the treatment of CRC. Camptothecin specifically binds to topoisomerase I, thereby inducing reversible single-strand breaks and unwinding the DNA double-stranded structure. IRI and its active metabolite SN-38 can bind to the topoisomerase I-DNA complex and prevent the religation of the cleaved single strands.^[4]^ Several clinical studies have demonstrated that IRI leads to significant improvement of CRC resistance to fluorouracil-based therapy.^[5–7]^ The most common toxicities associated with IRI are alopecia and gastrointestinal disturbances.^[8]^ OXA is a new platinum derivative used in the treatment of CRC. OXA acts on DNA by forming alkylated conjugates, which then form intrachain and interchain crosslinks, thereby inhibiting DNA synthesis and replication.^[9]^ The results of phase II/III trials showed that the OXA regimen has significant superiority over other regimens.^[8]^ Paresthesia and neurological disturbances are the most common toxicities in patients treated with OXA.^[8]^

In recent years, the eyes of the worldwide medical community have turned toward targeted molecular therapies.^[10]^ Bevacizumab is a recombinant humanized monoclonal antibody against vascular endothelial growth factor (VEGF) and is combined with standard chemotherapy to treat patients with mCRC.^[10]^ Bevacizumab acts directly on VEGF and blocks VEGF from binding to its receptor, which inhibits tumor angiogenesis, as well as tumor growth and metastasis.^[11]^ Previous studies suggested a significant improvement in the median progression-free survival (PFS) and overall survival (OS) in the bevacizumab arm.^[11]^ At present, IRI plus bevacizumab and OXA plus bevacizumab are the most common combination therapies for mCRC.^[12]^ A pivotal phase II trial demonstrated that IRI and OXA combined with a bevacizumab regimen has promising activity in patients with mCRC.^[13]^ In addition, several clinic trials have indicated significant improvement in terms of response rate, PFS, and OS.^[14,15]^

Several meta-analyses of randomized controlled trials (RCTs) showed that OXA has better curative effects than IRI.^[16,17]^ In the past 5 years, some new clinical trials that compared the difference between the 2 groups have emerged, but comparisons of IRI with bevacizumab and OXA with bevacizumab were not performed in these studies. This meta-analysis therefore aimed to compare the efficacy and safety between IRI ± bevacizumab and OXA ± bevacizumab in patients with mCRC. We are aware that more studies are required to better guide the treatment of patients with mCRC.

## 2 Materials and methods

### 2.1 Search strategy

The meta-analysis was performed using a systematic assessment according to the Preferred Reporting Items for Systematic Reviews and Meta-analysis guidelines.^[18]^ PubMed, EMBASE, and the Cochrane Controlled Trials Register were searched to identify relevant original articles written in English and published until December 2018. Various combinations of terms were searched, including “colorectal cancer,” “colon cancer,” “rectal cancer,” “oxaliplatin,” “irinotecan,” “bevacizumab,” and “randomized controlled trial.”

### 2.2 Selection criteria

To be eligible for our analysis, studies had to meet the following inclusion criteria: include patients with mCRC; patients in the treatment arm were exposed to IRI ± bevacizumab regimens, and patients in the control arm were exposed to OXA ± bevacizumab regimens; the treatment arm and control arm were compared without confounding by additional agents or interventions (ie, in the combination chemotherapy group, the treatment and control arms had to differ only by the IRI and OXA components); and RCTs.

Two reviewers independently assessed all the identified abstracts for inclusion using a standardized form with eligibility criteria. If the eligibility of the abstract was unclear, the full text of the article was retrieved for clarification. Each study was fully examined to eliminate duplicates.

### 2.3 Data assessment and quality assessment

Two reviewers subjectively reviewed all studies and independently extracted data from the studies. All disagreements were discussed until a consensus was reached. The following information was extracted: first author, year of publication, country, type of cancer, chemotherapy regimen, the number of patients, OS, time to progression (TTP), overall response rate (ORR), and toxicity. The hazard ratios (HRs) for OS and TTP were extracted directly from the original studies or were estimated indirectly from the survival curves.^[19]^ The quality of each study was assessed according to the methods described by the Cochrane Collaboration tool.^[18]^

### 2.4 Statistical analysis

The primary comparisons were between OS and TTP, while the secondary endpoints included evaluations of ORR and toxicity. The relevant effect measures (OS, PFS, TTP) of the HRs and 95% confidence intervals (CIs) were extracted directly from the original studies or were estimated indirectly from the survival curves. The Cochran Q or the *I*^2^ statistic was used to evaluate heterogeneity. Significant heterogeneity was considered present for *P* (Q) < .1 or *I*^2^ >50%. If heterogeneity existed, the random-effects model was used to evaluate the data.^[20]^ Otherwise, the fixed-effects model was applied due to a lack of significant heterogeneity.^[20]^ The presence of publication bias was evaluated through funnel plots using Begg and Egger tests, and all statistical analyses were calculated using the STATA version 14.0 software (Stata Corporation, College Station, TX).^[21,22]^ A *P*-value < .05 was considered to indicate a statistically significant difference between the 2 groups.

## 3 Results

### 3.1 Description of included trials

**Figure 1:**
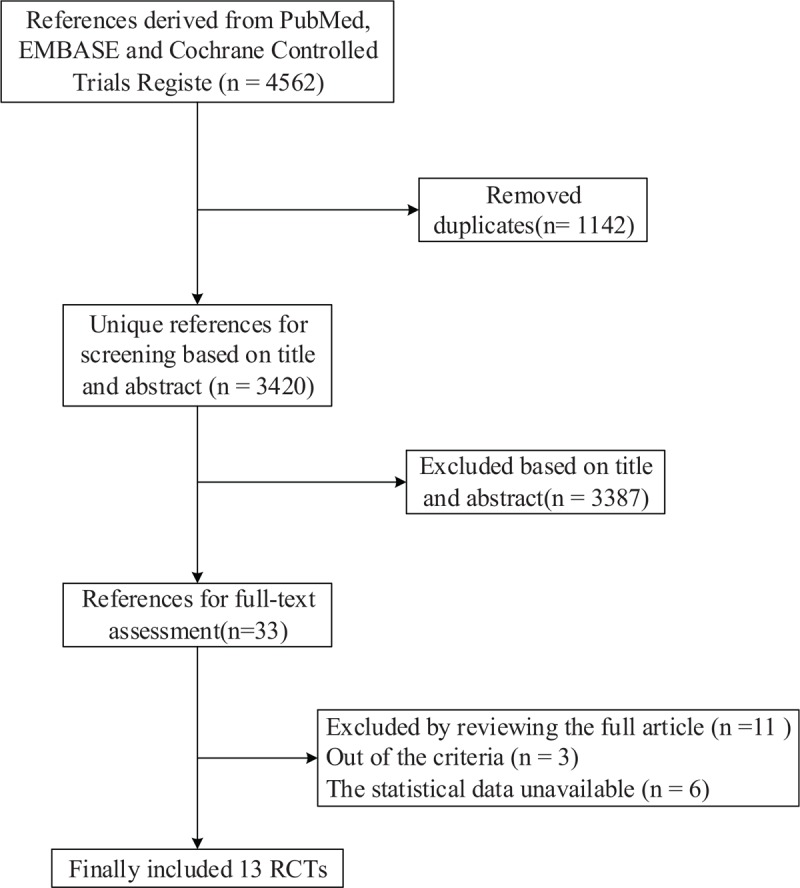
Flow diagram summarizing the search strategy. Thirteen randomized controlled trials (RCTs) were included the meta-analysis. No differences were found in the baseline characteristics between patients in the 2 groups in the selected studies.

This meta-analysis included 13 studies,^[8,13–15,23–31]^ and all selected studies were RCTs. The inclusion and exclusion criteria of the studies are shown in the flow diagram in Fig. 1. In all, 4191 patients with mCRC were included in the primary analysis. Among these patients, 2092 patients received IRI ± bevacizumab regimens, and 2099 patients were exposed to OXA ± bevacizumab regimens. The baseline characteristics of the included studies are summarized in Table 1. No differences were found in the baseline characteristics between patients in the 2 groups in the selected studies.

**Table 1:**
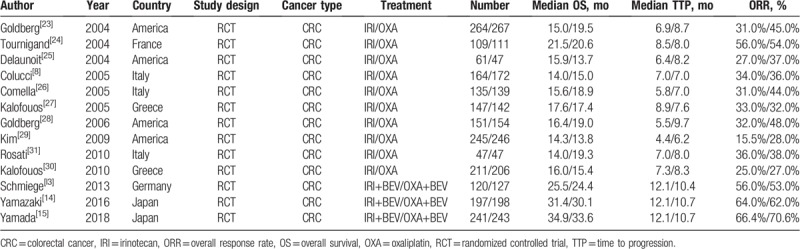
Characteristics of literatures included in the meta-analysis.

### 3.2 Overall survival (OS)

**Figure 2:**
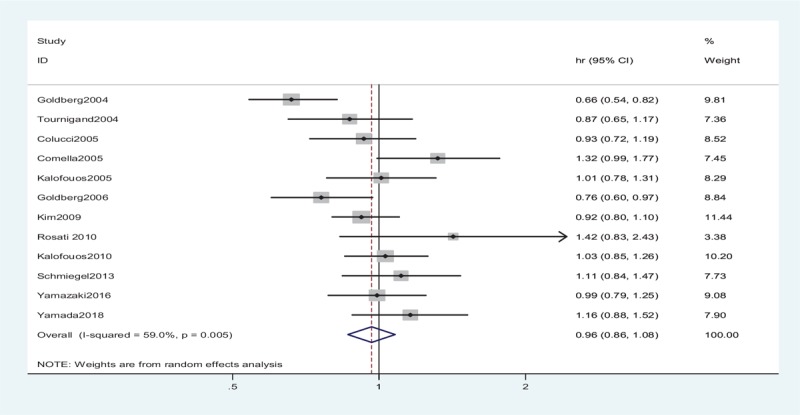
Random-effects model of HR (95% CI) of overall survival associated with the irinotecan group compared with the oxaliplatin group. There are no striking heterogeneity in overall survival was found (*P* = .005, *I*^2^ = 59.0%) in the studies. A random-effects model was applied to the meta-analysis. No significant differences were observed in the overall survival between the 2 arms (*P* = .53). CI = confidence interval, df = degrees of freedom, HR = hazard ratio.

The OS data were reported in the 13 trials, but only 6 studies^[13–15,23,28,29]^ included the relevant effect measures of the HRs and 95% CIs in the original articles. The HRs and 95% CIs in the other studies^[8,24–27,30,31]^ were estimated indirectly from the survival curves. No striking heterogeneity in OS was found (*P* = .005, *I*^2^ = 59.0%) in the 12 studies. Therefore, a random-effects model was applied to the meta-analysis. No significant differences were observed in the OS between the 2 arms (HR = 0.96, 95% CI: 0.86–1.08, *P* = .53) (Fig. 2, Table 2).

**Table 2:**
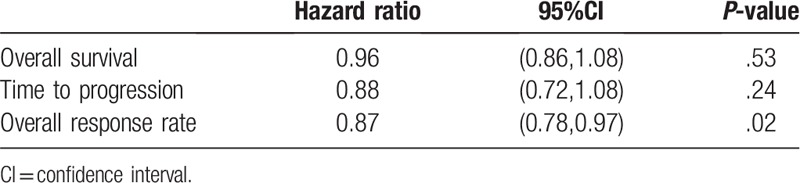
Efficacy of irinotecan group compared with oxaliplatin group in all treated patients.

### 3.3 Time to progression (TTP)

**Figure 3:**
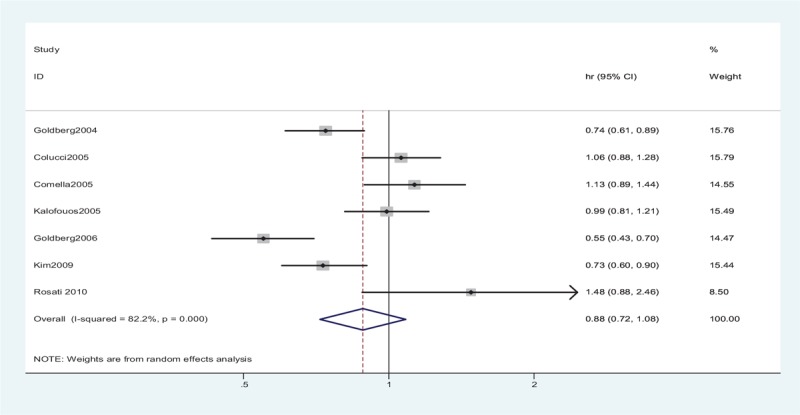
Random-effects model of HR (95% CI) of time to progression associated with the irinotecan group compared with the oxaliplatin group. No striking heterogeneity was found in the time to progression (*P* < .001, *I*^2^ = 82.2%) in studies. A random-effects model was applied to the meta-analysis. No significant differences were observed in TTP between the 2 groups (*P* = .24). CI = confidence interval, df = degrees of freedom, HR = hazard ratio, TTP = time to progression.

The TTP data were reported in 8 studies. However, only 3 studies^[23,28,29]^ included the relevant effect measures of HRs and 95% CIs in the original articles. The HRs and 95% CIs in the other studies^[8,25–27,30]^ were estimated indirectly from the survival curves. No striking heterogeneity was found in the TTP (*P* < .001, *I*^2^ = 82.2%) in 7 studies. Therefore, a random-effects model was applied to the meta-analysis. No significant differences were observed in TTP between the 2 groups (HR = 0.88, 95% CI: 0.72–1.08, *P* = .24) (Fig. 3, Table 2).

### 3.4 Overall response rate (ORR)

**Figure 4:**
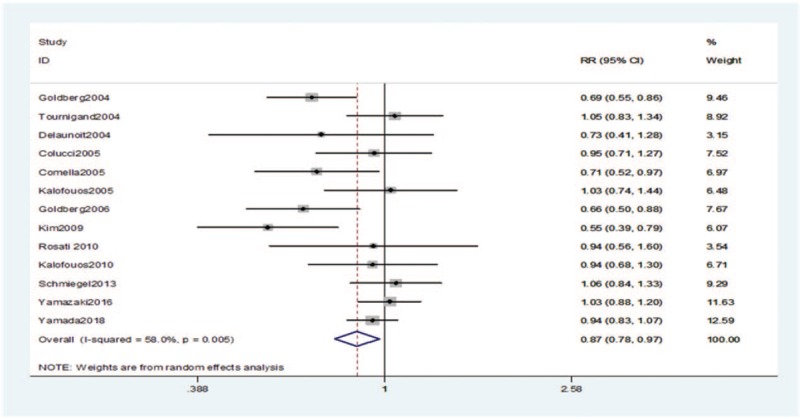
RR (95% CI) of overall response rate associated with the irinotecan group compared with the oxaliplatin group. No striking heterogeneity in overall response rate was found (*P* = .005, *I*^2^ = 58.0%) in the studies. A random-effects model was applied to the meta-analysis. The overall response rate was inferior in patients who received irinotecan ± bevacizumab compared with those who received oxaliplatin ± bevacizumab (*P* = .02). CI = confidence interval, df = degrees of freedom, RR = risk ratio.

The ORR data were available in 13 studies. No striking heterogeneity in ORR was found (*P* = .005, *I*^2^ = 58.0%) in the studies. For patients with mCRC, the ORR was inferior in patients who received IRI ± bevacizumab compared with those who received OXA ± bevacizumab (risk ratio [RR] = 0.87, 95% CI: 0.78–0.97, *P* = .02) (Fig. 4, Table 2).

### 3.5 Toxicities

Adverse effects were reported in all included studies (Table 3). The results revealed that the incidences of grade 3/4 nausea (RR = 1.63, 95% CI: 1.28–2.07, *P* < .001), vomiting (RR = 1.40, 95% CI: 1.09–1.81, *P* = .009), diarrhea (RR = 1.44, 95% CI: 1.23–1.70, *P* < .001), and anemia (RR = 4.13, 95% CI: 2.75–6.22, *P* < .001) were higher in the IRI arm. However, the incidences of grade 3/4 neutropenia (RR = 0.75, 95% CI: 0.68–0.83, *P* < .001), thrombocytopenia (RR = 0.43, 95% CI: 0.26–0.73, *P* = .002), and paresthesia/neurological disturbances (RR = 0.04, 95% CI: 0.02–0.07, *P* < .001) were higher in the OXA arm.

**Table 3:**
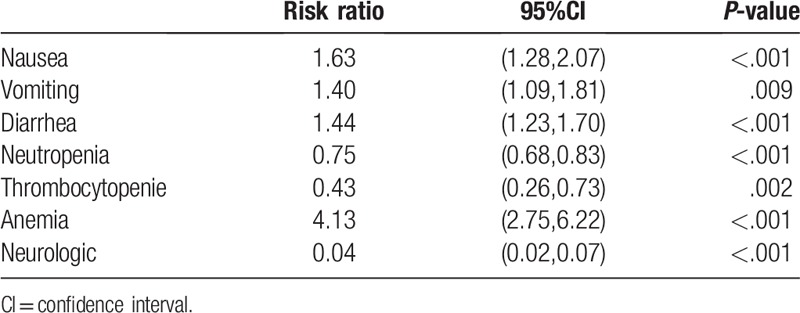
Grade 3 or 4 adverse events of irinotecan group compared with oxaliplatin group in all treated patients.

### 3.6 Publication bias

**Figure 5:**
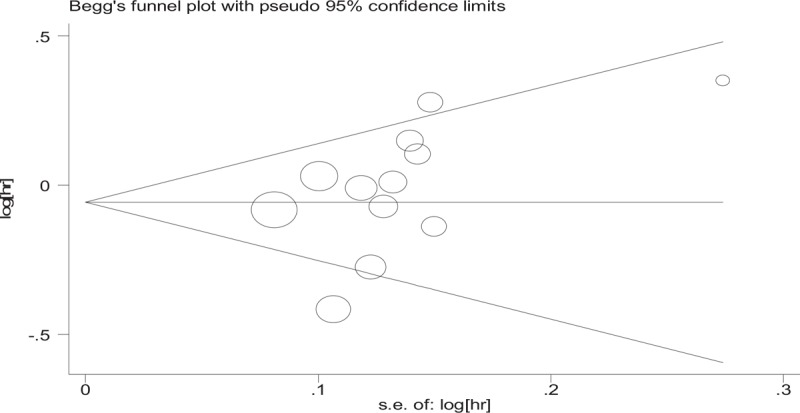
Begg funnel plots of publication bias test: overall survival. There are no striking heterogeneity in overall survival was found (*P* = .005, *I*^2^ = 59.0%) in the studies. The shapes of the funnel plots suggested that the included studies did not have any publication bias. HR = hazard ratio, se = standard error.

Publication bias in the literature was assessed by constructing Begg funnel plot and performing Egger test. The shapes of the funnel plots suggested that the included studies did not have any publication bias (Fig. 5). The symmetry of the funnel plots was statistically verified by Egger test. The results also did not indicate any evidence of publication bias.

## 4 Discussion

Although significant improvements were not observed in OS and TTP, our meta-analysis indicated that OXA ± bevacizumab was associated with a superior ORR compared with IRI ± bevacizumab in patients with mCRC. Therefore, OXA ± bevacizumab should be preferred as a first-line treatment for patients with advanced CRC. The study by Zhuang et al analyzed the efficacy of IRI and OXA regimens in the treatment of advanced CRC.^[16]^ The results demonstrated that OXA was superior to IRI in terms of both OS and TTP. In addition, the trial conducted by Liang et al showed that OXA significantly prolonged survival and was associated with lower toxicity.^[17]^ From the perspective of efficacy, our conclusions were consistent with the findings of previous meta-analyses.

In addition, some contrasting results may have confounded the findings and are worthy of further discussion. Some studies demonstrated that no significant difference was observed between the 2 groups in terms of ORR.^[8,14,15,24,25,27,30,31]^ However, a substantial improvement in ORR in the OXA ± bevacizumab group was eventually observed after the data were pooled, although a significantly longer median OS and TTP were reported in 4 previous trials.^[23,26,28,29]^ Our meta-analysis suggested that OXA ± bevacizumab as a maintenance treatment did not result in any significant improvement in OS and TTP. According to the comprehensive analyses, the errors of individual clinical trials were avoided, and we were able to draw a reliable conclusion. Accordingly, we concluded that the OXA ± bevacizumab regimen would be accepted as a standard treatment option for patients with mCRC.

IRI is a semisynthetic camptothecin derivative that is a specific topoisomerase I inhibitor. IRI has been approved for the treatment of advanced CRC in Europe since 1995.^[4]^ OXA is a platinum-based drug, which does not have the same anticancer spectrum as cisplatin. However, OXA is still effective for patients with CRC who have failed cisplatin treatment.^[9]^ With the emergence of novel targeted biologic therapies, the treatment of mCRC has evolved significantly over the past 2 decades.^[32]^ The survival rate has improved during this time and is approximately double that of 2 decades ago.^[32]^ Bevacizumab is a monoclonal antibody directed against VEGF and is combined with standard chemotherapy for the treatment of mCRC.^[10]^ By inhibiting the action of VEGF, bevacizumab can restrain the proliferation of endothelial cells and prevent the development of new blood vessels, thereby inhibiting tumor growth and metastasis.^[32]^ IRI with bevacizumab and OXA with bevacizumab are currently the most common combination therapies for mCRC. However, the direct comparison of IRI + bevacizumab and OXA + bevacizumab had not yet been performed. Our meta-analysis revealed that OXA ± bevacizumab had similar curative effects to IRI ± bevacizumab in terms of OS and TTP. However, the OXA regimen led to a remarkable improvement in ORR. The differences were more significant in clinical trials, in which patients were treated with the 2 chemotherapy drugs plus bevacizumab.^[13–15]^ Furthermore, more clinical trials are required to confirm these inconsistent conclusions.

Several clinical trials suggested that gastrointestinal and hematological toxicities were frequent in patients treated with IRI or OXA.^[23]^ In addition, the most common toxicities of OXA were related to neurologic disturbances. Previous studies indicated that bevacizumab is associated with a high risk of adverse events, including hypertension, proteinuria, and bleeding,^[33]^ which is associated with its inhibitory effect on VEGF.^[34,35]^ In general, the 2 treatment regimens showed some manageable adverse effects. Our results revealed that the incidences of gastrointestinal side effects (nausea, vomiting, and diarrhea) and anemia were more pronounced in the IRI group than in the OXA group. However, the occurrence of neutropenia, thrombocytopenia, and neurologic disturbances was significantly higher among patients treated with OXA ± bevacizumab. Other adverse effects, including mucositis, hair loss, and proteinuria were mentioned in only a subset of RCTs, were not further evaluated. These adverse effects occurred less frequently during treatment and could be statistically analyzed. From the perspective of toxicity, our conclusions were consistent with those of previous meta-analyses.^[16,17]^

This meta-analysis has some limitations that are expected to be improved in the future. First, the different combinations of chemotherapy regimens and doses may have led to limitations in a given meta-analysis. Second, some RCTs had a small sample size, which may have affected the final analysis.^[31]^ The study by Rosati et al required patients to be older than 70 years, which was relatively unusual compared with other studies.^[31]^ Furthermore, not all patients with mCRC received OXA or IRI as a first-line treatment in RCTs. For example, in the trial conducted by Kim et al, patients received OXA or IRI as a second-line therapy.^[29]^ More importantly, clinical data on the treatment of CRC with IRI + bevacizumab or OXA + bevacizumab are relatively rare, and only 3 RCTs met the requirements. As the number of clinical trials increases, we should continue to perform meta-analyses to compare the efficacy and safety of IRI plus bevacizumab and OXA plus bevacizumab.

Our meta-analysis compared the efficacy and safety of all RCTs over the past 20 years in which patients with mCRC were treated with OXA and IRI. The inclusion of a large number of studies was conducive to forming reliable conclusions. We not only compared the difference in efficacy between OXA and IRI, but we also compared the difference in efficacy between each of those 2 chemotherapy drugs alone and the 2 drugs combined with bevacizumab. This meta-analysis had an accurate conclusion, which suggests that the results can better guide clinical practice. As the number of clinical trials continues to increase, we will continue to analyze and summarize the differences in efficacy and safety between OXA and IRI in the future.

## 5 Conclusion

No statistically significant differences were observed in OS and TTP. However, the OXA group was superior to the IRI group in terms of ORR. Moreover, the 2 treatment regimens showed manageable toxicities. The results suggested the superior efficacy of OXA ± bevacizumab therapy compared with IRI ± bevacizumab therapy for mCRC patients.

## Acknowledgments

The authors thank the National Natural Science Foundation of China (81572928, 81772978 and 81773516) and the Jiangsu Provincial Special Program of Medical Science (BL2012016) for the support.

## Author contributions

**Conceptualization:** Jiali Dai, Dongying Gu, Jinfei Chen.

**Data curation:** Jiali Dai, Yuetong Chen, Yang Gong, Wenjing Zhao.

**Formal analysis:** Jiali Dai, Yuetong Chen, Yang Gong, Jingsun Wei, Xiaowen Cui, Wenjing Zhao.

**Funding acquisition:** Jinfei Chen, Dongying Gu.

**Investigation:** Jiali Dai, Hualin Yu, Jinfei Chen.

**Methodology:** Jiali Dai, Yuetong Chen, Yang Gong, Jingsun Wei, Xiaowen Cui, Hualin Yu, Dongying Gu, Jinfei Chen.

**Project administration:** Dongying Gu, Jinfei Chen.

**Software:** Jiali Dai, Yuetong Chen, Yang Gong, Jingsun Wei, Wenjing Zhao.

**Supervision:** Dongying Gu, Jinfei Chen.

**Validation:** Dongying Gu, Jinfei Chen.

**Visualization:** Xiaowen Cui, Hualin Yu, Dongying Gu, Jinfei Chen.

**Writing – original draft:** Jiali Dai, Yuetong Chen.

**Writing – review & editing:** Jiali Dai, Dongying Gu, Jinfei Chen.
